# Differentiating central nervous system demyelinating disorders: The role of clinical, laboratory, imaging characteristics and peripheral blood type I interferon activity

**DOI:** 10.3389/fphar.2022.898049

**Published:** 2022-08-12

**Authors:** Dimitris K. Karathanasis, Anna Rapti, Adrianos Nezos, Charalampos Skarlis, Constantinos Kilidireas, Clio P. Mavragani, Maria Eleftheria Evangelopoulos

**Affiliations:** ^1^ First Department of Neurology, School of Medicine, Eginition Hospital, National and Kapodistrian University of Athens, Athens, Greece; ^2^ Department of Physiology, School of Medicine, National and Kapodistrian University of Athens, Athens, Greece; ^3^ Fourth Department of Internal Medicine, School of Medicine, University Hospital Attikon, National and Kapodistrian University of Athens, Haidari, Greece; ^4^ Joint Academic Rheumatology Program, School of Medicine, National and Kapodistrian University of Athens, Athens, Greece

**Keywords:** type I interferon score, multiple sclerosis, systemic autoimmune disease, MS-like, demyelination, neuropsychiatric lupus, sjögren’s syndrome, systemic lupus erythematosus

## Abstract

**Objective:** While multiple sclerosis (MS) is considered the cornerstone of autoimmune demyelinating CNS disorders, systemic autoimmune diseases (SADs) are important MS mimickers. We sought to explore whether distinct clinical, laboratory, and imaging characteristics along with quantitation of peripheral blood type I interferon (IFN) activity could aid in differentiating between them.

**Methods:** A total of 193 consecutive patients with imaging features suggesting the presence of CNS demyelinating disease with or without relevant clinical manifestations underwent full clinical, laboratory, and imaging evaluation, including testing for specific antibodies against 15 cellular antigens. Expression analysis of type I IFN-inducible genes (MX-1, IFIT-1, and IFI44) was performed by real-time PCR, and a type I IFN score, reflecting type I IFN peripheral activity, was calculated. After joint neurological/rheumatological evaluation and 1 year of follow-up, patients were classified into MS spectrum and CNS autoimmune disorders.

**Results:** While 66.3% (n = 128) of the patients were diagnosed with MS spectrum disorders (predominantly relapsing–remitting MS), 24.9% (n = 48) were included in the CNS autoimmune group, and out of those, one-fourth met the criteria for SAD (6.7% of the cohort, n = 13); the rest (18.1% of the cohort, n = 35), despite showing evidence of systemic autoimmunity, did not fulfill SAD criteria and comprised the “demyelinating disease with autoimmune features” (DAF) subgroup. Compared to the MS spectrum, CNS autoimmune patients were older, more frequently females, with increased rates of hypertension/hyperlipidemia, family history of autoimmunity, cortical dysfunction, anti-nuclear antibody titers ≥1/320, anticardiolipin IgM positivity, and atypical for MS magnetic resonance imaging lesions. Conversely, lower rates of infratentorial and callosal MRI lesions, CSF T2 oligoclonal bands, and IgG-index positivity were observed in CNS autoimmune patients. Patients fulfilling SAD criteria, but not the DAF group, had significantly higher peripheral blood type I IFN scores at baseline compared to MS spectrum [median (IQR)]: 50.18 (152.50) vs. −0.64 (6.75), *p*-value: 0.0001.

**Conclusion:** Our study suggests that underlying systemic autoimmunity is not uncommon in patients evaluated for possible CNS demyelination. Distinct clinical, imaging and laboratory characteristics can aid in early differentiation between MS and CNS-involving systemic autoimmunity allowing for optimal therapeutic strategies. Activated type I IFN pathway could represent a key mediator among MS-like-presenting SADs and therefore a potential therapeutic target.

## Introduction

Though multiple sclerosis (MS) is considered the cornerstone of the central nervous system (CNS) demyelinating disorders, several clinical entities are important MS mimickers and need to be taken into consideration in the differential diagnosis (Brownlee et al., 2017; Thompson et al., 2018a; Thompson et al., 2018b; Solomon et al., 2019). Among those, systemic autoimmune diseases (SADs), neuromyelitis optica spectrum disease (NMOSD), and myelin oligodendrocyte glycoprotein antibody-associated disease (MOGAD) are the most common. Less frequently, neurosarcoidosis, neuro-Behçet disease, and chronic lymphocytic inflammation with pontine perivascular enhancement responsive to steroids (CLIPPERS) may present with MS reminiscent manifestations. Despite a distinct pathogenetic background, atherosclerotic small vessel disease or even cerebral autosomal dominant arteriopathy with subcortical infarcts and leukoencephalopathy (CADASIL), Fabry disease, and Susac’s syndrome can also present with MS-resembling features (Thompson et al., 2018b).

SADs, mainly systemic lupus erythematosus (SLE), Sjögren’s syndrome (SS), and antiphospholipid syndrome (APS) have been previously shown to invariably affect the CNS, with transverse myelitis, optic neuritis, and white matter hyper-intensities (WMH) in CNS magnetic resonance imaging (MRI) being the main MS-like manifestations (Delalande et al., 2004; Ferreira et al., 2005; Pelidou et al., 2007; Theodoridou and Settas, 2008; Massara et al., 2010; Magro Checa et al., 2013; Piga et al., 2017; Hughes, 2018; Schwartz et al., 2019). In previous studies, higher rates of homozygous methylenetetrahydrofolate reductase (MTHFR) mutations have been found among patients with autoimmune features and MS-like or vasculitic CNS disease, and a link has been identified between anti-thyroid antibodies with nonspecific WMH and CNS involvement in the setting of APS (Mavragani et al., 2007; Mavragani et al., 2009).

While usually helpful, brain MRI (Stosic et al., 2010; Akasbi et al., 2012; Magro Checa et al., 2013; Magro-Checa et al., 2018) and cerebrospinal fluid (CSF) analysis (Govoni et al., 2016; Venhoff et al., 2019) cannot consistently differentiate between MS and CNS involvement in the setting of SADs. Though the detection of serum autoantibodies has a central role in the diagnosis of SAD, they are reported to precede disease onset by many years (Arbuckle et al., 2003). Additionally, low-titer antinuclear (ANA) and antiphospholipid antibodies are frequently found in the context of MS at variable frequencies ranging between 3%-–63% and 0.9%–8.5%, respectively (Spadaro et al., 1999; Szmyrka-Kaczmarek et al., 2012; Malyavantham et al., 2015; Merashli et al., 2017). Of interest, MRZ reaction [detection of CSF IgG antibody indices to measles (M), rubella (R), and varicella-zoster (Z)] was found to be prevalent in patients with MS and ANA positivity compared to those with rheumatological disease with CNS involvement, providing a potential differentiating tool between the two entities (Venhoff et al., 2019).

Despite the potential phenotypic similarities, distinct underlying pathogenetic mechanisms and therapeutic approaches characterize MS and SAD with CNS involvement. For instance, activation of type I interferon (IFN) pathway has been considered a central pathogenetic event in SADs in association with antibodies against ribonucleoproteins, disease activity indices, and renal involvement (Baechler et al., 2003; Kirou et al., 2005; Weckerle et al., 2011; Tsokos et al., 2016; Ronnblom and Leonard, 2019; Postal et al., 2020; Idborg and Oke, 2021), with type I IFN receptor blockade being recently approved for lupus patients (Morand et al., 2020; Deeks, 2021).

Compared to lupus, MS patients display 30-fold lower type I IFN serum activity (Feng et al., 2012), while type I IFN-induced gene expression has been shown to vary among relapsing–remitting MS (RRMS) patients (van Baarsen et al., 2006). Moreover, a subgroup of MS patients with high peripheral blood type I IFN signature, was found to be refractory to exogenous administration of recombinant IFN-β (Comabella et al., 2009), a well-established MS treatment (Author anonymous, 1993; Kieseier, 2011). Along the same lines, the latter has been shown to exacerbate lupus or other SADs (Borg and Isenberg, 2007; Airo et al., 2008; Bahri et al., 2012).

In view of these diagnostic and therapeutic challenges, in the current study, we sought to explore whether clinical, laboratory, and imaging characteristics as well as type I IFN inducible gene expression in peripheral blood could differentiate MS from systemic autoimmune diseases presenting with CNS involvement. Furthermore, we sought to investigate potential associations between activation of type I IFN pathway with distinct clinical, serological, and imaging features.

## Patients and methods

### Patients

This was a prospective study, evaluating consecutive patients presenting with imaging features suggesting the presence of demyelinating disease with or without relevant clinical manifestations. After initial neurological assessment and the exclusion of ischemic/hemorrhagic cerebrovascular events, space-occupying lesions, or acute CNS infections, patients with MRI findings raising the suspicion of CNS demyelinating disease were referred to the Demyelinating Diseases Unit of the First Department of Neurology of the National and Kapodistrian University of Athens (NKUA) Medical School at Eginition Hospital for further diagnostic evaluation. Thus, we enrolled 205 consecutive patients from 1 January 2019 to 31 March 2021. The study was approved by the Eginition Hospital Ethical Committee and prior to study enrollment, all patients provided written informed consent, in accord with the requirements of the declaration of Helsinki (Author anonymous, 2013).

Exclusion criteria included the previous diagnosis of MS or SAD, concurrent active infection, and treatment with immunosuppressive or corticosteroids over the last 3 months. Therefore, our final study sample consisted of 193 patients, which underwent full clinical, laboratory, and imaging evaluation. Demographics (sex and age), clinically relevant co-morbidities, and family history of MS or systemic/organ-specific autoimmune disease (AD) were recorded. Neurological manifestations were classified according to the CNS region affected (cortex, optic nerve, brainstem, pyramidal/sensory tracts, cerebellum, and bowel/bladder function) by an experienced neurologist (M.E.E). Bilateral optic neuritis, other visual disturbances, headache, transient loss of consciousness, tinnitus, speech disturbance, and isolated fatigue were classified as features nontypical for MS (Brownlee et al., 2017; Thompson et al., 2018a; Solomon et al., 2019). Evaluation for systemic autoimmune features in the presence of autoantibody positivity was performed by an experienced rheumatologist (C.P.M) and included assessment of constitutional, musculoskeletal and mucocutaneous symptoms, sicca complaints, Raynaud’s phenomenon, cardiorespiratory manifestations, and gastrointestinal/renal abnormalities.

### Laboratory Evaluation

Standard laboratory evaluation at baseline included complete blood count, coagulation tests [including prothrombin time (PT) and activated partial thromboplastin time (aPTT)], chemistry panel, B12, and 25-OH-D3 vitamin levels, thyroid function tests, protein electrophoresis, and urinalysis, as well as testing for chronic viral infections [human immunodeficiency virus (HIV) and hepatitis B/C]. Immunology profile testing was also performed including rheumatoid factor (RF), complement levels, antinuclear antibodies (ANA), anti-double strand DNA (anti-dsDNA), and antiphospholipid antibodies, as well as antibodies against cellular antigens. Anti-MOG and anti-aquaporin-4 (AQP4) antibodies were tested upon clinical indication. CSF was obtained by a lumbar puncture at baseline and tested for cell count, glucose and protein levels, oligoclonal bands (OCBs), and IgG index (cutoff of ≥0.65 for positive values, as set by the Laboratory Department of Eginition Hospital). The presence of OCBs was detected with isoelectric focusing (IEF) on agarose gels followed by immunofixation, one of the two most sensitive techniques for this purpose (Freedman et al., 2005). The procedure included isoelectrofocusing on an agarose gel to fractionate the proteins in the CSF and serum samples, performed on the semi-automatic HYDRASYS system (SEBIA, France) followed by immunofixation with anti-IgG antiserum. CSF findings were compared directly with findings from serum samples run simultaneously in the same assay in an adjacent track. Finally, the IgG immunofixation patterns of CSF and serum from the same patient were visually compared and categorized into five OCB constellation types (1–5), according to the European consensus study (Andersson et al., 1994). The evaluation was performed by one experienced clinical pathologist. The presence of ANA was assessed by indirect immunofluorescence (IIF) on the substrate from human Hep-2 larynx cancer cell culture, while antiphospholipid and anti-double strand DNA antibodies were measured by an enzyme-linked immunosorbent assay (ELISA). In isolated sera from all enrolled patients, we tested for auto-antibodies against 15 cellular antigens (RNP/Sm, Sm, SS-A (Ro-60), Ro-52, SS-B, Scl-70, PM-Scl-100, Jo-1, centromere protein B, PCNA, dsDNA, nucleosomes, histones, ribosomal P-proteins, and AMA M), using the EUROLINE ANA Profile 3 commercial immunoblot kit (EUROIMMUN Medizinische Labordiagnostika AG, Germany), as part of a wholesome diagnostic approach. According to the manufacturer’s instructions for signal intensity, results for each of the above autoantibodies were either: negative (signal <6), borderline (6–10), medium (11–25), or strong (>25) positive. Borderline results were classified as negative.

### Imaging Evaluation

MRI studies of the CNS were performed in all enrolled patients (in 68.1% of patients within 30 days from clinical evaluation; for the rest, the median timespan was 3 months). Magnetic fields of 1.5 or 3 T, including at least T1-weighted, T2-weighted, fluid-attenuated inversion recovery (FLAIR), and T1 post-gadolinium sequences for brain studies were implemented. Based on clinical criteria, at least one spinal cord (cervical or thoracic) MRI study was performed in the majority of patients, including at least short-tau inversion recovery (STIR), T2-weighted and T1-weighted pre- and post-gadolinium sequences. MRI data were analyzed blindly to both the clinical and laboratory features of the patients. White matter lesions were classified according to CNS localization and imaging characteristics (Filippi et al., 2016; Filippi et al., 2019). More specifically, classification according to localization was based on lesion distribution in periventricular, juxtacortical/cortical/deep white matter (essentially all non-periventricular, supratentorial lesions), infratentorial, corpus callosum, optic nerve, and spinal cord (cervical and/or thoracic) areas. On the basis of the imaging characteristics, the presence of gadolinium enhancement and characteristic morphology (size>3mm, ovoid shape, long axis vertical orientation toward the corpus callosum/ventricles) points toward lesions “typical” for MS (Filippi et al., 2016; Filippi et al., 2019; Solomon et al., 2019). Tumefactive and Balo lesions were also considered typical MS lesions. Nonspecific WMH and small vessel disease were classified as atypical for MS radiographic lesions (Supplementary Table S1).

### Final diagnosis

Upon thorough clinical and laboratory evaluation at 1 year of follow-up patients received a final diagnosis, as follows:

a. MS spectrum disorders [RRMS, primary progressive MS (PPMS), secondary progressive MS (SPMS), clinically isolated syndromes (CIS), radiologically isolated syndromes (RIS), and tumefactive MS and MOGAD](Thompson et al., 2018a).
b. CNS autoimmune group, including patients with clinical and/or laboratory features suggestive of a systemic autoimmune disease either fulfilling previously published classification criteria (SAD with CNS involvement) (Miyakis et al., 2006; Shiboski et al., 2017; Antunes et al., 2018; Aringer et al., 2019a; Aringer et al., 2019b) or not [demyelinating disease with autoimmune features (DAF)], as previously suggested (Nikolopoulos et al., 2021).
c. Non autoimmune small vessel disease (MRI WMH of presumed vascular origin, not typical for MS, in the absence of clinical and serological systemic autoimmune features) (Wardlaw et al., 2019).
d. Other (no evidence of MS, CNS autoimmune disorder, or small vessel disease).

### Quantitation of peripheral blood type I IFN score

Total RNA was extracted from freshly isolated, available, whole peripheral blood of 144 patients by TRIzol Reagent (ThermoFisher Scientific, United States). The quantity and quality of RNA samples were spectrophotometrically tested (Biospec Nano, Japan). One microgram of total RNA was reverse-transcribed into cDNA with PrimeScript™ RT Reagent Kit (Perfect Real Time) (TAKARA, Japan). Samples were 1:10 diluted in nuclease-free water and stored at −20°C. Quantitative real-time polymerase chain reaction (qRT-PCR) was performed in order to quantify mRNA expression of the following genes, preferentially induced by type I IFN: myxovirus (influenza virus) resistance 1 (MX-1), IFN-induced protein with tetratricopeptide repeats 1 (IFIT-1), and IFN-induced protein 44 (IFI44). As an internal control and normalization gene, glyceraldehyde phosphate dehydrogenase (GAPDH) was used. All reactions were performed in duplicate. A reference sample was included in each PCR plate, to ensure normalization across experiments. Peripheral blood type I IFN score was calculated as previously described (Kirou et al., 2004; Nezos et al., 2015). In detail, mean and standard deviation (SD) levels of each IFN inducible gene (IFIG) in 15 healthy controls (HC) consistently used as standards in our lab were used to standardize expression levels of each gene for each study subject using the following formula (RE IFIG subject—Mean HC)/SD HC. The standardized expression levels were subsequently summed for each patient to provide a type I IFN score as the sum of each study subject’s relative expression for each of the three genes preferentially induced by type I IFN. According to a cut-off value defined as the mean plus three SD of the corresponding IFN scores in HC (8.50), patients were further divided into high and low IFN groups.

### Statistical Analysis

Continuous and categorical variables were assessed by Mann–Whitney and chi-square tests, respectively. Continuous variables are presented as mean ± SD or median and interquartile range (IQR). Statistical analysis was performed using the IBM^®^ SPSS^®^ software platform and GraphPad Prism^®^ software. Differences were considered significant when *p*-value was <0.05.

## Results

### Demographics, clinical, laboratory, and imaging characteristics of the study cohort

Table 1 summarizes the demographical, clinical, laboratory, and imaging characteristics of the study cohort. Among 193 patients included in the final study sample, 139 (72.0%) were females and their age was (mean ± SD): 40.2 ± 12.7 years, with sensory, pyramidal tracts, and optic nerve being the main CNS affected areas (35.5%, 26.9%, and 23.1%, respectively). According to MRI, demyelinating brain or spine lesions were detected in 68.6% of patients, while 26.2% had nonspecific (nontypical for MS) WMH. In the entire study cohort, the prevalence of type 2 OCBs in CSF was 49.7%, with the corresponding figure being 2.5% for both 3 and 4 types. Half of the patients with type 3 OCBs were eventually diagnosed with MS spectrum disorders with the other half being classified in the CNS autoimmune group (Thompson and Freedman, 2006; Tumani et al., 2020). Furthermore, 41.3% of study participants had a CSF IgG Index ≥0.65. As for their immune profile, 8.5% of the patients enrolled had ANA titers ≥1/320 and 17.0% were positive for antiphospholipid antibodies. Antibodies against MOG and AQP4 were detected in 2.3% and 1.6% of those tested, respectively.

**TABLE 1:** Descriptive characteristics of the 193 study participants.

Demographics	
Sex (female) (%)	72.0
Age (years) (mean ± SD)	40.2 ± 12.7
Age of Onset (years) (mean ± SD)	37.8 ± 12.2
Family history(%)	—
MS	10.4
SAD	9.1
Organ Specific Autoimmune Disease	14.1
Comorbidities(%)	
Organ-Specific Autoimmune Disease^a^	17.8
Diabetes Mellitus	5.0
Hypertension	8.3
Hyperlipidemia	14.3
Clinical characteristics	
First manifestation (%)	—
Optic neuritis	23.1
Brainstem	14.5
Pyramidal tracts	26.9
Cerebellar	10.8
Sensory tracts	35.5
Bowel/Bladder	3.8
Symptoms nontypical for MS^b^	18.8
MRI Findings	
Demyelinating brain or spine lesions (%)	68.6
Nonspecific brain WMH (%)	26.2
Nonspecific brain WMH with demyelinating spine lesions (%)	8.9
Areas affected in MRI (%)	—
Cortical/juxtacortical/deep white matter (non-periventricular supratentorial)	74.2
Periventricular	79.8
Infratentorial	50.3
Optic Nerve	10.2
Corpus Callosum	41.7
Cervical Spine	53.6
Thoracic Spine	45.7
Gadolinium enhancement at current evaluation^c^(%)	38.2
CSF Findings	
T2 Positive OCBs (%)	49.7
High IgG Index (≥0.65) (%)	41.3
White cell count (pof) (mean ± SD)	5.1 ± 7.1
CSF protein (mg/dl) (mean ± SD)	39.5 ± 17.9
Autoantibodies	
ANA (titers ≥1/320) (%)	8.5
Antiphospholipid antibody positivity (%)	
Any	17.0
Anticardiolipin IgM	15.7
Anticardiolipin IgG	0.0
Anti-β2GPI IgM	4.7
Anti-β2GPI IgG	1.8
Anti-MOG (%)	2.3
Anti-AQP4 (%)	1.6

a Hashimoto thyroiditis, psoriasis, vitiligo, and hidradenitis suppurativa

b dizziness, bilateral ON, visual disturbances other than ON, headache, cervical or low back pain, sensory symptoms without a CNS pattern, transient loss of consciousness, tinnitus, speech disturbance, isolated fatigue, and cortical features (aphasia, encephalopathy, cognitive impairment, and seizures)

c MRIs with gadolinium administration performed during the last 30 days

Abbreviations ANA = anti-nuclear antibodies, AQP4 = aquaporin-4, CNS = central nervous system, CSF = cerebrospinal fluid, Ig = immunoglobulin, MRI = magnetic resonance imaging, MOG = myelin oligodendrocyte glycoprotein, MS = multiple sclerosis, ON = optic neuritis, OCBs = oligoclonal bands, SAD = systemic autoimmune disease, WMH = white matter hyperintensities, and β2GPI = beta-2-glycoprotein-I

### Final diagnosis at 1 year of follow-up

As shown in Table 2, after 1 year of follow-up, 66.3% (n = 128) of all study participants were diagnosed with MS spectrum disorders, with RRMS being the most prevalent (43.0%). Furthermore, 24.9% (n = 48) had evidence of CNS involvement in a background of systemic autoimmunity (CNS autoimmune group) and they were further divided into those fulfilling SAD classification criteria (6.7%, n = 13) and those classified as DAF (18.1%, n = 35). The SAD diagnoses were: SLE: 2.1% (n = 4), SS: 3.6% (n = 7), and APS: 0.5% (n = 1) and undifferentiated connective tissue disease (UCTD): 0.5% (n = 1). Of interest, one patient with SS also had anti-AQP4 antibodies. Finally, 14 out of 193 patients (7.3%) were classified as non-autoimmune small vessel disease, while 1.6% (n = 3) had no evidence of MS, CNS autoimmune disease, or small vessel disease and were classified as “other.”

**TABLE 2:** Final diagnoses in the 193 patients of the study cohort at 1 year of follow-up.

Final diagnosis	
MS Spectrum disorders [n (%)]	128 (66.3)
RRMS	83 (43.0)
PPMS	6 (3.1)
SPMS	4 (2.1)
CIS	18 (9.3)
RIS	10 (5.2)
Tumefactive MS	6 (3.1)
MOGAD	1 (0.5)
CNS autoimmune group [n (%)]	48 (24.9)
DAF [n (%)]	35 (18.1)
SAD [n (%)]	13 (6.7)
Primary SS	7 (3.6)
SLE	4 (2.1)
APS	1 (0.5)
UCTD	1 (0.5)
Non-autoimmune small vessel disease [n (%)]	14 (7.3)
Other* [n (%)]	3 (1.6)

* No evidence of MS, CNS autoimmune disorder, or small vessel disease

Abbreviations APS = antiphospholipid syndrome, CIS = clinically isolated syndrome, CNS = central nervous system, DAF = demyelinating disease with autoimmune features, MS = multiple sclerosis, MOGAD = myelin oligodendrocyte glycoprotein antibody disease, PPMS = primary progressive multiple sclerosis, RIS = radiologically isolated syndrome, RRMS = relapsing–remitting multiple sclerosis, SAD = systemic autoimmune disease, SLE = systemic lupus erythematosus, SPMS = secondary progressive multiple sclerosis, SS = Sjögren’s syndrome, and UCTD = undifferentiated connective tissue disease

### Comparative analysis of demographical, clinical, laboratory, and imaging characteristics between MS spectrum and CNS autoimmune groups

A comparative analysis between MS spectrum and CNS autoimmune groups, regarding demographics, family history, and co-morbidities, as well as clinical, imaging, and laboratory characteristics are presented in Table 3. Compared to MS spectrum disorders, patients with autoimmune features, fulfilling or not criteria for SAD diagnosis (CNS autoimmune group), had higher rates of female sex (85.4% vs. 66.4%, *p*-value: 0.013), as well as of positive family history for SAD and organ-specific AD (22% vs. 5.4%, *p*-value: 0.002; 24.4% vs. 10.9%, *p*-value: 0.037, respectively). At the same time, MS spectrum patients were younger both at the time of evaluation [median age (IQR): 37.0 (16.8) vs. 46.1 (26.5), *p*-value: <0.001] and onset of neurological symptoms [median age (IQR): 34.8 (16.6) vs. 43.0 (20.6), *p*-value 0.003]. Moreover, both hypertension and hyperlipidemia were more prevalent in the CNS autoimmune group (17.1% vs. 5.3%, *p*-value: 0.019 and 28.6% vs. 6.1%, *p*-value: <0.001, respectively). In terms of first clinical manifestation, a statistically significant predominance of cortical dysfunction was observed in CNS autoimmune patients versus the MS spectrum group (8.9% vs. 0%, *p*-value: 0.001).

**TABLE 3:** Comparative analysis between MS spectrum and CNS autoimmune groups.

	MS Spectrum Disease (n = 128)	CNS Autoimmune Group (n = 48)	*p*-value
Demographics			
Female sex (%)	66.4	85.4	0.013
Age (years) [median (IQR)]	37.0 (16.8)	46.1 (26.5)	<0.001
Age of Onset (years) [median (IQR)]	34.8 (16.6)	43.0 (20.6)	0.003
Family History (%)		
MS	10.8	9.8	0.851
SAD	5.4	22.0	0.002
Organ-specific autoimmune disease	10.9	24.4	0.037
Comorbidities (%)		
Diabetes	4.4	2.4	0.564
Hypertension	5.3	17.1	0.019
Hyperlipidemia	6.1	28.6	<0.001
First manifestation (%)		
Optic neuritis	23.0	31.1	0.283
Brainstem	18.3	6.7	0.063
Pyramidal	30.2	24.4	0.467
Cerebellar	11.9	4.4	0.151
Sensory	37.3	33.3	0.635
Bladder/bowel	3.2	4.4	0.691
Cortical	0	8.9	0.001
MRI findings (%)		
Demyelinating brain or spine lesions	85.7	63.9	0.003
Demyelinating spine lesions	71.8	58.8	0.150
Nonspecific brain WMH	10.2	44.7	<0.001
Nonspecific brain WMH with demyelinating spine lesions	7.3	23.5	0.007
Areas affected in MRI (%)		
Cortical/juxtacortical/deep hite matter (non-periventricular supratentorial)	73.4	73.9	0.945
Periventricular	84.3	75.6	0.192
Infratentorial	58.1	37.8	0.020
Optic nerve	9.5	15.6	0.269
Corpus callosum	51.0	28.9	0.020
Cervical spine	61.7	48.6	0.160
Thoracic spine	50.7	43.5	0.546
Gadolinium enhancement (%)		
Any enhancement in MRIs performed during the last 30 days	44.4	40.5	0.653
CSF findings		
T2 positive OCBs (%)	60.7	37.1	0.036
High IgG index (%)	47.4	27.3	0.04
CSF white cell count (pof) [median (IQR)]	3.0 (6.0)	2.0 (5.0)	0.089
CSF protein (mg/dl) [median (IQR)]	35.5 (18.3)	35.5 (23.0)	0.741
Laboratory parameters		
ANA titers ≥1/320 (%)	2.6	27.3	0.0001
ANA titers: 1/160 (%)	8.5	18.2	0.08
Anti-cardiolipin IgG positivity (%)	0	0	1.0
Anti-cardiolipin IgM positivity (%)	12.9	27.9	0.03
Anti-β2GPI IgG positive (%)	1.7	2.4	0.05
Anti-β2GPI IgM positive (%)	4.3	7.7	0.68
C3 (mg/dl) [median (IQR)]	109.0 (36.8)	111.5 (25.8)	0.479
C4 (mg/dl) [median (IQR)]	23.0 (6.8)	25.0 (13.0)	0.348
White blood cells (per μl) [median (IQR)]	6900.0 (2500.0)	6600.0 (1890.0)	0.301
Hemoglobin (g/dl) [median (IQR)]	14.0 (2.0)	13.5 (0.5)	0.769
Platelets (10^3^ per μl) [median (IQR)]	262.0 (84.0)	262.0 (92.8)	0.654
ESR (mm/hr) [median (IQR)]	8.0 (7.3)	8.5 (13.8)	0.428
Folic acid (μg/L) [median (IQR)]	6.0 (2.3)	9.5 (7.8)	0.610
Vitamin B12 (ng/L) [median (IQR)]	368.5 (183.3)	480.0 (298.5)	0.053
25-OH-vitamin-D (ng/ml) [median (IQR)]	25.0 (9.0)	24.0 (7.0)	0.445
Total cholesterol (mg/dl) [median (IQR)]	192.0 (35.0)	215.0 (42.0)	0.083
LDL (mg/dl) [median (IQR)]	109.0 (45.5)	137.0 (51.5)	0.131
HDL (mg/dl) [median (IQR)]	58.0 (17.0)	64.0 (16.0)	0.262
TG (mg/dl) [median (IQR)]	87.0 (49.0)	91.0 (40.0)	0.705
Abnormal urinalysis (%)	0	0	

Abbreviations ANA = antinuclear antibodies, CNS = central nervous system, CSF = cerebrospinal fluid, ESR = erythrocyte sedimentation rate, HDL = high density lipoprotein, Ig = immunoglobulin, IQR = interquartile range, LDL = low density lipoprotein, MRI = magnetic resonance imaging, MS = multiple sclerosis, OCBs = oligoclonal bands, SAD = systemic autoimmune disease, TG = triglycerides, WMH = white matter hyperintensities, and β2GPI = beta-2-glycoprotein-I

Regarding imaging findings, demyelinating brain or spine lesions characterized predominantly the MS spectrum patients (85.7% vs. 63.9%, *p*-value: 0.003), whereas atypical for MS brain lesions (nonspecific WMH) prevail in the CNS autoimmune group (44.7% vs. 10.2%, *p*-value: <0.001). As for the affected CNS areas, only the infratentorial and corpus callosum lesions were significantly more frequent in MS spectrum disease (58.1% vs. 37.8%, and 51.0% vs. 28.9%, respectively, with *p*-value: 0.02 for both comparisons). CSF analysis revealed a higher prevalence of T2 OCBs in MS spectrum compared to the CNS autoimmune group (60.7% vs. 37.1%, *p*-value: 0.036), with similar results for high IgG index (47.4% vs. 27.3%, *p*-value: 0.04). Among the blood and urine laboratory parameters, ANA titers equal to or greater than 1/320, contrary to those equal to 1/160, were significantly more prevalent in the CNS autoimmune group compared to the MS spectrum group (27.3% vs. 2.6%, *p*-value: 0.0001 and 18.2% vs. 8.5%, *p*-value: 0.08, respectively). Moreover, CNS autoimmune patients had higher rates of anti-cardiolipin IgM and anti-beta-2-glycoprotein-I (β2GPI) IgG antibodies (27.9% vs. 12.9%, *p*-value: 0.03 and 2.4% vs. 1.7%, *p*-value: 0.05, respectively). Another worth-mentioning trend was that of lower levels of B12 vitamin in MS spectrum patients [median (IQR): 368.5 (183.3) vs. 480.0 (298.5), *p*-value: 0.05].

The SAD and DAF groups were very similar in all of the above characteristics, with no statistically significant differences between them (data not shown). Notably, anti-Ro/SSA (Ro52 and Ro60) were the most frequently detected specific autoantibodies among SAD patients (data not shown).

### Type I IFN Score

**FIGURE 1:**
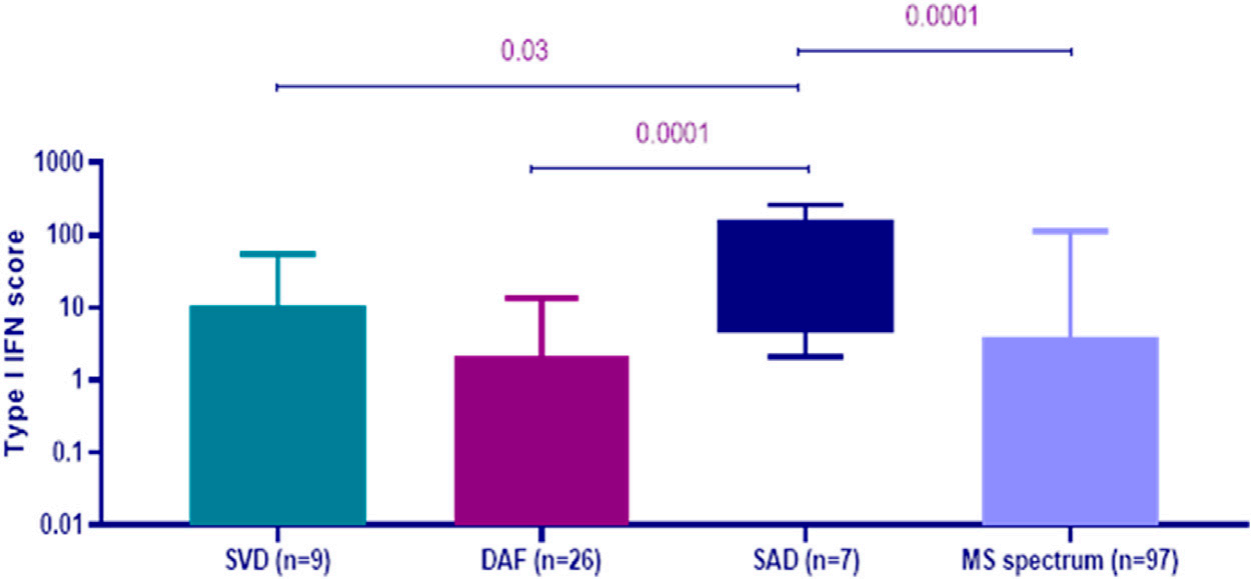
Peripheral blood type I interferon (IFN) scores at first evaluation according to the final diagnosis at 1 year of follow-up. Patients with systemic autoimmune disease (SAD) with central nervous system (CNS) involvement (n = 7) had higher type I IFN score at baseline [median (IQR): 50.18 (152.50)] compared to those with small vessel disease (SVD, n = 9) [median (IQR): 0.68 (12.56), p-value: 0.03], demyelinating disease with autoimmune features (DAF, n = 26) [median (IQR): 0.23 (4.40), p-value: 0.0001] and multiple sclerosis (MS) spectrum disorders (n = 97) [median (IQR): 0.64 (6.75), p-value: 0.0001]. Error bars represent the minimum and maximum of data distribution.

As displayed in Figure 1, peripheral blood type I IFN activity at baseline, as reflected by type I IFN score, was found to be significantly higher in patients, who were ultimately classified as SAD with CNS involvement [median (IQR): 50.18 (152.50)] compared to those within the MS spectrum group [median (IQR): 0.64 (6.75), *p*-value: 0.0001], DAF [median (IQR): 0.23 (4.40), *p*-value: 0.0001], and small vessel disease [median (IQR): 0.68 (12.56), *p*-value: 0.03]. No other significant differences were detected between groups.

### Clinical, laboratory, and imaging features in high vs. low type I IFN groups

To further explore whether type I IFN peripheral blood activity may influence demographical, clinical, imaging, and laboratory characteristics, a comparative analysis between high and low type I IFN groups was performed (Table 4).

**TABLE 4:** Clinical, imaging and laboratory features in high vs. low type I IFN groups (See Methods for classification).

	High IFN (n = 26)	Low IFN (n = 116)	*p* value
Demographics			
Female sex (%)	92.3	66.4	0.008
Age (years) [median (IQR)]	40.9 (24.9)	37.7 (17.6)	0.450
Age of onset (years) [median (IQR)]	35.9 (17.5)	36.2 (17.5)	0.847
Family History (%)		
MS	5.3	13.9	0.298
Systemic/Organ-specific autoimmune disease	21.1	28.0	0.531
Comorbidities (%)		
Hypertension	4.5	9.8	0.431
Hyperlipidemia	13.6	12.9	0.923
Current clinical evaluation (%)		
Active optic neuritis	9.1	17.7	0.317
Brainstem	18.2	34.8	0.126
Pyramidal tracts	81.8	69.6	0.247
Cerebellar	40.9	29.5	0.290
Sensory tracts	50.0	37.5	0.273
Bowel/Bladder	18.2	15.2	0.723
MRI findings (%)		
Demyelinating brain or spine lesions	69.6	77.0	0.449
Nonspecific brain WMH	13.0	7.1	0.346
Nonspecific brain WMH with demyelinating spine lesions	17.4	8.9	0.225
Areas affected in MRI (%)		
Cortical/juxtacortical/deep white matter (non-periventricular supratentorial)	91.7	68.4	0.020
Periventricular	91.7	80.2	0.181
Infratentorial	45.8	57.5	0.295
Optic Nerve	12.5	9.6	0.664
Corpus Callosum	44.4	46.8	0.854
Cervical spine	60.9	54.7	0.590
Thoracic spine	53.8	45.3	0.574
Gadolinium enhancement (%)		
Any enhancement during the last 30 days	41.7	45.7	0.718
CSF findings (%)		
Positive T2 OCBs	50.0	47.6	0.852
High IgG Index	35.3	37.6	0.854
Immune profile (%)		
ANA titers ≥1/320	21.7	4.6	0.01
Any specific autoantibody^a^, medium positive	11.5	16.2	0.548
Any specific autoantibody^a^, strong positive	15.4	5.1	0.064
Anti-cardiolipin IgM	13.0	17.0	0.643
Anti-cardiolipin IgG	0	0
Anti-β2GPI IgM	4.8	4.7	0.986
Anti-β2GPI IgG	4.5	1.9	0.443
Laboratory parameters [median (IQR)]		
White blood cells (per μl)	5850.0 (2825.0)	6900.0 (2300.0)	0.064
Neutrophils (per μl)	3500.0 (2010.0)	4000.0 (2225.0)	0.047
Lymphocytes (per μl)	1850.0 (1231.8)	2000 (800)	0.225
Platelets (10^3^ per μl)	229.0 (93.0)	267.5 (88.5)	0.088

a anti-nRNP/Sm, anti-Sm, anti-SSA/Ro60, anti-Ro52, anti-SSB/La, anti-Scl70, anti-PM-Scl-100, anti-CENP B, anti-PCNA, anti-dsDNA, anti-nucleosomes, anti-histones, anti-AMA-M2, anti-Jo1, and anti-ribosomal P

Abbreviations ANA = anti-nuclear antibodies, CSF = cerebrospinal fluid, IFN = interferon, Ig = immunoglobulin, IQR = interquartile range, MRI = magnetic resonance imaging, MS = multiple sclerosis, OCBs = oligoclonal bands, WMH = white matter hyperintensities, and β2GPI = beta-2-glycoprotein-I

Female sex was more prevalent in 92.3% of high IFN patients versus 66.4% of low IFN subjects (*p*-value: 0.008). Except for cortical/juxtacortical/deep white matter (non-periventricular) lesions, which were more frequently detected in patients with high type I IFN scores (91.7% vs. 68.4%, *p*-value: 0.02), no other significant association was detected between any other imaging findings or neurological symptom with type I IFN scores. Moreover, high type I IFN scores were associated with ANA titers ≥1/320 (21.7% in high-IFN group vs. 4.6% in the low-IFN group, *p*-value: 0.01), as well as with lower neutrophil levels [median (IQR): 3500.0 (2010.0) vs. 4000.0 (2225.0) per μl, *p*-value: 0.047)]. Of note, three out of five patients with high IFN scores within the SAD group had positive serum titres of anti-Ro/SSA antibodies (data not shown).

## Discussion

In the present study, we enrolled consecutive, undiagnosed, and treatment-naive patients with MS-like disease presentation, with the aim to explore whether distinct clinical, serological, CSF, and imaging features, as well as baseline peripheral blood type I IFN activity could aid in the formulation of a final diagnosis after 1 year of follow-up. Out of the 193 patients, included in our cohort, two-thirds were classified as MS spectrum disease and one-fourth as CNS autoimmune group. Among the latter, one-fourth met the criteria for SAD (predominantly SS and SLE), while the rest did not fulfill SAD criteria, constituting the DAF subgroup. In comparison to MS spectrum, CNS autoimmune patients displayed distinctive characteristics; they were older, more frequently females, more prone to suffer from hypertension and/or hyperlipidemia, and more likely to report a positive family history of autoimmunity. Cortical dysfunction as the first neurological presentation, atypical for MS MRI lesions, as well as less frequent T2 OCBs and IgG index positivity along with higher rates of ANA titers ≥1/320 and anti-cardiolipin IgM and anti-β2GPI IgG positivity also characterized the CNS autoimmune group. Moreover, peripheral blood type I IFN score at baseline was shown to be significantly elevated only in patients fulfilling classification criteria for SAD compared to all other groups. Of note, patients with high type I IFN scores were mainly females, and had increased rates of cortical/juxtacortical/deep white matter (non-periventricular) supratentorial lesions and ANA titers ≥1/320, together with lower neutrophil counts.

Our study suggests that underlying systemic autoimmunity is not infrequent in undiagnosed patients undergoing diagnostic evaluation for possible demyelinating CNS disease. In line with previous studies, stronger female predominance, frequent sparing of the corpus callosum, and preferential involvement of cortical/juxtacortical/deep white matter (non-periventricular) regions compared to the periventricular area have been well described in the context of neuropsychiatric SLE versus MS, with the exact opposite being true for the cerebellum, brainstem, and basal ganglia (Magro Checa et al., 2013). Moreover, hypertension and dyslipidemia were more prevalent in the CNS autoimmune group in accordance with previous comparative studies reporting a pronounced risk for cardiovascular events in lupus and rheumatoid arthritis compared to MS patients (Setyawan et al., 2021). Though lower ANA titers and antiphospholipid antibody positivity have been extensively reported in MS and MS-like populations (Spadaro et al., 1999; Szmyrka-Kaczmarek et al., 2012; Malyavantham et al., 2015; Merashli et al., 2017), in the current report, we emphasize the role of higher ANA titers (more than 1/320) pointing toward an underlying systemic autoimmune process, in support of previous findings (Magro Checa et al., 2013). Moreover, in agreement with previous works, a higher CSF IgG index and the presence of OCBs reinforce the diagnosis of MS without however ruling out a diagnosis of SAD (Magro Checa et al., 2013; Govoni et al., 2016; Venhoff et al., 2019). Of interest, MS spectrum patients displayed lower B12 vitamin levels in comparison to their CNS autoimmune counterparts, possibly due to malabsorption mediated by autoimmune reactivity toward gastrointestinal antigens commonly found in MS populations (Banati et al., 2013).

Of note, lupus and primary SS were the prevailing SAD diagnoses detected in our cohort, compatible with previous observations (Delalande et al., 2004; Bertsias and Boumpas, 2010; Massara et al., 2010; Unterman et al., 2011; Borowoy et al., 2012; Schwartz et al., 2019) and anti-Ro/SSA were the predominant reactivity among patients with SAD. This is in accord with previous studies on lupus and primary SS also highlighting the role of anti-Ro/SSA as a marker of neuropsychiatric involvement (Alexander et al., 1994; Mikdashi and Handwerger, 2004; Morreale et al., 2014; Fan et al., 2021; Tetsuka et al., 2021) and disease activity in the setting of NMOSD (Lin et al., 2022).

It is also intriguing that peripheral blood type I IFN activity, consistently found to be associated with anti-Ro/SSA antibodies in both lupus and SS patients (Kirou et al., 2005; Weckerle et al., 2011; Mathian et al., 2019), was a strong predictor for SAD diagnosis among patients with presentation suspicious of CNS demyelinating disease included in the current cohort. This is consistent with the work by Yusof et al. suggesting that an IFN score based on quantitation of type I and II IFN inducible gene expression in peripheral blood along with the family history of autoimmunity was able to predict the conversion to SAD in ANA-positive individuals (El-Sherbiny et al., 2018; Md Yusof et al., 2018).

Furthermore, the demyelinating disease with autoimmune features (DAF) group, displaying a low type I IFN score in peripheral blood, may represent a distinct clinical entity deserving close follow-up and tailored diagnostic and therapeutic approaches (Mavragani et al., 2007; Nikolopoulos et al., 2021). Interestingly, a recent study, described that almost half of the patients presenting with demyelinating lesions and coexisting clinical and/or serological evidence of autoimmunity, not fulfilling criteria for either MS or SAD at baseline, will also continue to lack a well-defined diagnosis after a median follow-up period of 3 years (Nikolopoulos et al., 2021). As previously reported, specific autoantibodies such as anti-Ro/SSA can be present years prior to disease diagnosis (Arbuckle et al., 2003), while the emergence of a well-defined autoimmune disease is in parallel with an elevation of type I IFN activity in peripheral blood (Munroe et al., 2016). Therefore, the possibility of DAF switching to SAD in the future is thought to be substantial.

While nonspecific brain WMH, as well as hypertension and dyslipidemia, were significantly higher in the CNS autoimmune group with respect to MS, none of these characteristics could differentiate patients with high versus low IFN activity. Although WMH is the most frequently observed MRI lesion in patients with systemic autoimmune diseases and suspected CNS involvement (Magro Checa et al., 2013; Magro-Checa et al., 2018), our CNS autoimmune group mainly includes the MS-like disease patients with autoimmune features (DAF group), in which type I IFN was not upregulated in contrast to individuals meeting classification criteria for SAD. Since IFN signature surfaces in parallel with clinically overt disease (Munroe et al., 2016), one could hypothesize that WMH represents an early sign of systemic autoimmunity prior to type I IFN pathway activation and full-blown disease manifestation.

Though activation of type I IFN pathway has been postulated as a major effector mechanism for several clinical manifestations in patients with lupus and SS (Mavragani, 2017; Postal et al., 2020), there is limited data supporting a role in the pathogenesis of neuropsychiatric manifestations in both serum (Fragoso-Loyo et al., 2012) and gene expression studies (Baechler et al., 2003). Of interest, variations of three prime repair exonuclease 1 (TREX1) genes–an exonuclease involved in the clearance of endogenous nucleic acids (Stetson et al., 2008) have been previously detected in European SLE cases with neurological involvement (Namjou et al., 2011). Mutations of the TREX1 gene are a hallmark of Aicardi–Goutières syndrome (AGS) (Crow et al., 2015), characterized by a lupus reminiscent picture with high IFN-α in peripheral blood and CSF, white matter lesions, autoantibody positivity and cytopenia (Ramantani et al., 2010; Crow and Manel, 2015; Cuadrado et al., 2015; Cattalini et al., 2016). Besides, previous studies in SS and lupus patients, as well as in TREX1-deficient human neural cells revealed a role of L1 (long interspersed nuclear element-1) retroelements as major triggers of type I IFN production in both target tissues (Mavragani et al., 2018) and astrocytes, respectively (Thomas et al., 2017). Moreover, ubiquitin-specific peptidase 18 (USP18) deletion in young adult mouse models [normally downregulating signal transducer and activator of transcription 1 (STAT1) signaling], led to increased activation of downstream type I IFN signaling in white matter microglial cells causing microgliopathy (Goldmann et al., 2015). Of interest, abrogation of type I IFN signaling in the Rnaset2^−/−^ mice characterized by AGS-resembling leukoencephalopathy syndrome, led to complete resolution of CNS features (Kettwig et al., 2021).

In line with previous reports, associations of type I IFN signature with female sex (Panchanathan et al., 2010; Gabriele et al., 2021), neutropenia (Hall et al., 2015; Mathian et al., 2019; Chasset et al., 2020) or high ANA titers (Wither et al., 2017) were detected. Moreover, the link between non-periventricular lesions and high IFN scores might reflect the presence of an immune-mediated vascular injury as the underlying potential mechanism, as formerly suggested (Skeoch and Bruce, 2015; Spinelli et al., 2018; Filippi et al., 2019; Wardlaw et al., 2019; Sereti et al., 2020). In support of these findings, it has been previously shown that type I IFN inhibition in SLE patients blocked NET formation and restored cholesterol efflux capacity impairment, already shown to contribute to lupus-related atherosclerotic disease (Giannelou and Mavragani, 2017; Casey et al., 2021; O'Neil et al., 2019; Carlucci et al., 2018). Of note, among MS patients, IFN-β therapy was associated with dysregulated metabolic profiles and higher blood pressure (Sternberg et al., 2014).

The main strength of our study was the large number and extensive evaluation of undiagnosed, treatment-naïve individuals, presenting with an episode described as potentially demyelinating. As for limitations, quantitation of type I IFN score was performed in a subset of recruited patients and the final number of SAD patients was rather small to draw definite conclusions. Moreover, our patients were not tested for lupus anticoagulant (LAC); however, the initial aPTT measurement did not reveal time prolongation in any case (Tripodi and Chantarangkul, 2017).

Taken together, the identification of a prominent type I IFN signature among SAD patients with evidence of CNS involvement as the cardinal manifestation might imply a potentially significant therapeutic role of type I IFN receptor blockade, already licensed for the treatment of lupus (Morand et al., 2020; Deeks, 2021). Conversely, treatment with IFN-β in patients with a systemic autoimmune background might lead to disease exacerbation and therefore alternative therapeutic strategies such as B cell depletion should be considered (Evangelopoulos et al., 2017; Sabatino et al., 2019).

In conclusion, our study indicates that underlying systemic autoimmunity is a considerable possibility in patients undergoing evaluation for possible demyelinating CNS disease and distinct clinical, imaging and laboratory features can reliably differentiate CNS autoimmune from MS spectrum individuals in an everyday clinical practice setting. Moreover, activation of type I IFN pathway seems to be a key mediator among SAD patients presenting with MS-like disease and therefore a potential therapeutic target. Finally, a close follow-up is recommended for CNS autoimmune patients not yet fulfilling well-defined SAD classification criteria, given the heightened risk for future development of full-blown systemic autoimmune disease. Thorough studies exploring the molecular pathways governing the generation of diverse aspects of multifaceted CNS demyelinating disease would allow the design and institution of tailored and most appropriate therapeutic strategies.

## Data Availability

The original contributions presented in the study are included in the article/Supplementary Materials, further inquiries can be directed to the corresponding author.
