# Supplementary material for: Differentiating central nervous system demyelinating disorders: The role of clinical, laboratory, imaging characteristics and peripheral blood type I interferon activity
Source: Front Pharmacol. 2022 Aug 12;13:898049. doi: 10.3389/fphar.2022.898049 (PMC9412761; doi:10.3389/fphar.2022.898049)
Supplement: Supplementary file 1 [file Table1.docx]

**Supplementary Table 1.** The main MRI lesions assessed and their characteristics.

| **Classification of MRI white matter lesions** | |
| --- | --- |
| **Lesion localization** | **Lesion characteristics** |
| Non-periventricular  (*juxtacortical/cortical/deep white matter*) | Morphology  (*size>3mm, ovoid shape, long axis vertical orientation towards the corpus callosum/ventricles*) |
| Periventricular | Gadolinium enhancement |
| Infratentorial | Tumefactive |
| Corpus callosum | Balo |
| Optic nerve |  |
| Spinal cord  (*cervical and/or thoracic*) |  |
| **Abbreviations**  **MRI**= magnetic resonance imaging | |
